# Acute Stroke: Current Evidence-based Recommendations for Prehospital Care

**DOI:** 10.5811/westjem.2015.12.28995

**Published:** 2016-03-02

**Authors:** Nancy K. Glober, Karl A. Sporer, Kama Z. Guluma, John P. Serra, Joe A. Barger, John F. Brown, Gregory H. Gilbert, Kristi L. Koenig, Eric M. Rudnick, Angelo A. Salvucci

**Affiliations:** * University of California San Diego, Department of Emergency Medicine, San Diego, California; † EMS Medical Directors Association of California, California; ‡ University of California San Francisco, Department of Emergency Medicine, San Francisco, California; § Stanford University, Department of Emergency Medicine, Stanford, California; # University of California Irvine, Center for Disaster Medical Sciences, Orange, California

## Abstract

**Introduction:**

In the United States, emergency medical services (EMS) protocols vary widely across jurisdictions. We sought to develop evidence-based recommendations for the prehospital evaluation and treatment of a patient with a suspected stroke and to compare these recommendations against the current protocols used by the 33 EMS agencies in the state of California.

**Methods:**

We performed a literature review of the current evidence in the prehospital treatment of a patient with a suspected stroke and augmented this review with guidelines from various national and international societies to create our evidence-based recommendations. We then compared the stroke protocols of each of the 33 EMS agencies for consistency with these recommendations. The specific protocol components that we analyzed were the use of a stroke scale, blood glucose evaluation, use of supplemental oxygen, patient positioning, 12-lead electrocardiogram (ECG) and cardiac monitoring, fluid assessment and intravenous access, and stroke regionalization.

**Results:**

Protocols across EMS agencies in California varied widely. Most used some sort of stroke scale with the majority using the Cincinnati Prehospital Stroke Scale (CPSS). All recommended the evaluation of blood glucose with the level for action ranging from 60 to 80mg/dL. Cardiac monitoring was recommended in 58% and 33% recommended an ECG. More than half required the direct transport to a primary stroke center and 88% recommended hospital notification.

**Conclusion:**

Protocols for a patient with a suspected stroke vary widely across the state of California. The evidence-based recommendations that we present for the prehospital diagnosis and treatment of this condition may be useful for EMS medical directors tasked with creating and revising these protocols.

## INTRODUCTION

Each year, 795,000 people experience a new or recurrent stroke causing significant mortality and neurologic disability. 1 On average, every 40 seconds, someone in the United States has a stroke, accounting for one of every 18 deaths in the U.S.^1^ Emergency medical services (EMS) plays a pivotal role in recognizing acute strokes and providing timely transport to hospitals with specific stroke treatment capabilities. The optimal prehospital management and stroke system organization continue to evolve. EMS care varies widely across the U.S. The Institute of Medicine report, “Emergency Medical Services at the Crossroads,” notes that EMS needs more uniform high-quality care and specific standards for evaluating that care.^2^ One such standard is the prehospital protocol that EMS personnel follow while taking care of patients. Protocols vary widely between jurisdictions. We provide a summary of the evidence for the prehospital treatment of patients with suspected acute stroke and evaluate the consistency of California protocols.

## METHODS

The state of California divides EMS care into 33 local EMS agencies (LEMSAs). One set of governmental medical control policies regulates first responders and ambulance transporters in each county-wide or region-wide system. Medical directors of those agencies, along with other interested EMS medical directors, make up the EMS Medical Directors Association of California (EMDAC). EMDAC supports and guides the various agencies and makes recommendations to the California EMS Authority about policy, legislation and scope of practice issues. In an effort to improve the quality of EMS care in our state, EMDAC has endeavored to create evidence-based recommendations for EMS protocols. Those recommendations and previous reviews are intended to assist medical directors of the various LEMSAs to develop high quality, evidence-based protocols.

A subcommittee of EMDAC developed this manuscript and chose by consensus the elements that should be included in any protocol for a patient with a suspected acute stroke. The subcommittee then created a narrative review of the existing evidence for prehospital treatment of a patient with a suspected acute stroke. Clinical questions regarding those interventions were developed in the population, intervention, control and outcome (PICO) format. Our population included those patients in the prehospital setting with a suspected acute stroke. The intervention varied by clinical question. The control consisted of patients who were not receiving the specific intervention, and outcomes were defined by accuracy of diagnosis and neurologic or imaging outcome after intervention.

We relied heavily on recommendations made by various organizations that have performed systematic reviews and meta-analyses regarding treatment interventions including the American Heart Association (AHA) and the International Liaison Committee on Resuscitation (ILCOR). We supplemented the recommendations from those organizations with additional literature searches through PubMed from 1966 to 2015 for each question. The primary literature review of PubMed searched for the term “Prehospital and Stroke.” That yielded 476 articles, 86 of which were published in English, not review articles, and pertinent to the topics identified by the EMDAC subcommittee. That search was supplemented with additional PubMed searches for specific topics.

We assigned levels of evidence (LOE) and graded our recommendations based on the American College of Emergency Physicians (ACEP) process of creating their clinical policies with slight modification to better fit our objectives.^3^ This committee of EMDAC reviewed studies and assigned LOE based on the study design, including features such as data collection methods, randomization, blinding, outcome measures and generalizability. LOE I consisted of randomized, controlled trials, prospective cohort studies, meta-analysis of randomized trials or prospective studies or clinical guidelines/comprehensive review. LOE II consisted of nonrandomized trials and retrospective studies. LOE III consisted of case series, case reports, and expert consensus. After assigning LOE to the studies, we translated those to clinical grades of recommendations using the following standards:

### Level A Recommendations

Prehospital recommendations with a strong degree of certainty based on one or more LOE I studies or multiple LOE II studies.

### Level B Recommendations

Prehospital recommendations with a moderate degree of certainty based on one or more LOE II studies or multiple LOE III studies.

### Level C Recommendations

Prehospital recommendations based on only poor quality or minimal LOE III studies or based on consensus.

### No Recommendation

No recommendation was given in those cases where only preliminary data or no published evidence exists and we had no expert consensus. We also withheld recommendation when studies, no matter their LOE, showed conflicting data.

After answering the clinical question and providing recommendations for diagnostic and treatment interventions, we reviewed each current acute stroke protocol for the 33 agencies for consistency with the recommendations. The clinical protocols were reviewed during the month of June 2015. We deemed institutional review board approval not necessary for this review of publicly available research and clinical protocols.

### Use of a Stroke Scale

#### Clinical Question

Does the use of a prehospital stroke scale help identify strokes in patients found with acute neurological deficits, and which stroke scale is most effective?

#### Summary of Current Evidence

Timely recognition is the most critical step in the prehospital care of a patient with an acute stroke. Sepsis, hypo- or hyperglycemia, seizure, tumor, intracranial hemorrhage, migraine, and syncope can all cause acute neurological deficits. If a stroke is correctly identified, the patient can be appropriately transported to a designated stroke center that can provide timely care, including tissue plasminogen activator (tPA) or endovascular therapy when appropriate. The misdiagnosis of stroke may lead to delayed care or inappropriate treatment. When not recognized in the field, the initial triage process frequently misses the stroke.^4^ When EMS providers do not document a stroke scale, they are more likely to miss the diagnosis.^5^,^6^

There are many scoring systems to screen for an acute ischemic stroke in the field. EMS groups most commonly use Face Arm Speech Test (FAST), Cincinnati Prehospital Stroke Scale (CPSS) (most commonly used in California), or Los Angeles Prehospital Stroke Screen (LAPSS). Many of the more commonly used stroke scales are not designed to identify posterior circulation strokes.

FAST includes facial droop, arm weakness, speech difficulties, and time to seek medical help. FAST is simple to use and has shown reproducibility between physicians and paramedics.^7^ It had a sensitivity of 79–85% and specificity of 68%.^8^,^9^ However, FAST did not detect 38% of posterior cerebral circulation strokes.^7^

The CPSS includes three components – pronator drift, speech difficulties, and facial droop. Many studies have shown the reproducibility and validity of this scale between physicians and prehospital providers.^10^ Sensitivity ranged from 44–95% and specificity was 23–96%.^5^,^6^,^8^,^10^–^16^ With a score of two, it predicted patients receiving thrombolytic therapy with a 96% sensitivity and 65% specificity, although that has been studied less.^17^ The CPSS, like the FAST, is limited in that it was designed to identify middle cerebral artery strokes.

Recently, the Cincinnati Prehospital Stroke Severity Scale (CPSSS) was developed to predict severe anterior ischemic strokes and large vessel occlusions (LVO).^18^ Unlike the CPSS, the CPSSS grades the severity of the stroke. The scale gives two points for conjugate gaze deviation and one point for incorrectly answering at least one of two level of consciousness questions (age or current month).^18^ The scale further gives one point for not following at least one of two commands (close eyes, open and close hand) and one point for not holding an arm up for 10 seconds.^18^ CPSSS greater than or equal to two was 89% sensitive and 73% specific for National Institute of Health Stroke Scale (NIHSS) greater than or equal to 15, which predicts LVO.^18^ The recognition of LVOs may become more important as stroke systems develop and as advanced therapies show more efficacy.

The LAPSS is only used for patients over 45 years of age with an absence of history of seizure disorder, symptom duration less than 24 hours, and a blood glucose range of 60–400mg/dL.^19^ It detects unilateral weakness in facial grimace, handgrip and arm strength. 19 With those criteria, LAPSS was designed to decrease the false positive rate of CPSS. Paramedics using LAPSS demonstrated a sensitivity of 74–98%, with a specificity of 44–97%, PPV 86%, and NPV 98%.^8^,^11^,^12^,^19^,^20^

Los Angeles Motor Scale (LAMS) assigns values to the points on the LAPSS to assess severity, giving a score of zero through 10 with bilateral weakness or zero through five with unilateral weakness.^8^,^21^,^22^ LAMS quickly and effectively assesses for LVO. LAMS demonstrated a sensitivity of 81%, specificity of 89%, and accuracy of 85% for LVO if the LAMS score was four or higher.^21^ LAMS correlated closely with NIHSS and predicted three-month outcome.^22^

The Melbourne Ambulance Stroke Screen (MASS) includes speech difficulties plus the components of the LAPSS. In contrast with LAPSS, blood glucose range begins at 50mg/dL.^8^,^11^,^14^ Age must be greater than 45 years, and there must be no history of seizure or epilepsy. Patient must be ambulatory at baseline. Sensitivity was found to be as high as 83–98% with a specificity of 44–86% and 100% sensitivity for ischemic strokes eligible for thrombolytic therapy.^8^,^11^,^14^

If the patient does not have a history of seizures, symptom duration greater than 25 hours, or blood glucose outside 60–400mg/dL, the Medic Prehospital Assessment for Code Stroke (Med PACS) can rule in a stroke. Under those circumstances, it evaluates facial droop, gaze, arm and leg weakness and speech.^8^ Sensitivity ranged from 44–74% with specificity 32–98%.^8^,^16^

The Recognition of Stroke in the Emergency Room (ROSIER) score assesses facial, arm, or leg weakness, speech, and visual field deficits. Blood glucose must be >62mg/dL. Scores range from −2 to 5, with a score less than or equal to zero indicating a low likelihood of stroke. Seizure or syncope are scored as −1.^8^,^13^,^23^ It demonstrated a sensitivity of 80–89% and a specificity of 79–83%.^8^,^13^ Physicians confirmed 64% of strokes and 78% of non-strokes identified by ambulance clinicians with ROSIER.^23^

The most common scale used in the hospital setting is the NIHSS. For prehospital assessment, the shortened version was developed, including assessment of gaze, visual field, motor function of the right and left leg, language, level of consciousness, facial paresis, and dysarthria.^8^,^24^ It attempts to predict stroke severity but is more complicated than some of the other stroke scales. With its complexity, it can evaluate strokes outside of the middle cerebral artery distribution.

The Kurashiki Prehospital Stroke Scale (KPSS) is applied after a stroke is recognized by another stroke scale, such as the CPSS. It awards 13 points assessing consciousness, motor weakness, and speech.^8^,^25^–^29^ When used for recognition, sensitivity ranged from 83–86% and specificity ranged from 60–69% for detecting stroke.^8^ A KPSS score of 3–9 predicts candidates for tPA with a sensitivity of 84% and specificity of 93%.^26^ It is a simpler scale than the full NIHSS but showed good correlation with the NIHSS when used by emergency medical technicians and can predict long-term outcome.^25^,^28^,^29^

The Maria Prehospital Stroke Scale Score (MPSS) can be used both to identify strokes and to determine stroke severity. It grades facial droop, arm drift and speech disturbances, and the score predicts tPA use.^30^ The Rapid Arterial Occlusion Evaluation (RACE) scale is also based on the NIHSS to evaluate LVO via assessment of facial palsy, arm motor function, leg motor function, gaze, and aphasia or agnosia.^31^ The scale showed strong correlation with the NIHSS, sensitivity of 85%, and specificity of 68%.^31^

With the development of endovascular capable centers, the recognition of LVO may become more important, and the use of scales such as CPSSS, NIHSS, and KPSS may be useful in grading stroke severity and making destination decisions.

#### Current Prehospital Treatment Recommendation

##### Level A Recommendation

- A stroke scale should be used in the prehospital setting for any patient with an acute neurological deficit to rapidly assess and triage patients with possible stroke.
- There is currently no practical prehospital scale that accurately detects strokes outside of the middle cerebral artery distribution.
- CPSS and LAPSS are the most validated and most frequently used scales.

##### Level B Recommendation

- None given.

##### Level C Recommendation

- In the future, scales such as CPSSS, NIHSS and KPSS may be added to gauge stroke severity and direct transport to a higher level of care, e.g. comprehensive rather than basic stroke receiving center.

### Blood Glucose Evaluation

#### Clinical Question

Should paramedics measure glucose and administer dextrose in hypoglycemic patients in cases of suspected stroke?

#### Summary of Current Evidence

Hypo- and hyperglycemia both mimic stroke.^32^ It is critical to measure glucose levels when there is concern for a possible stroke. This will differentiate between stroke and hypoglycemia. Symptoms such as hemiparesis, hemiplegia, speech or visual disturbances, confusion, and poor coordination can all present in patients with hypoglycemia and can be corrected with administration of dextrose.^33^–^35^ While symptoms such as tremulousness and altered behavior may occur with milder degrees of hypoglycemia, focal stroke-like neurological symptoms, such as hemiplegia, typically do not manifest until glucose levels are less than 45mg/dL.^36^–^38^ There is a clear benefit to giving dextrose to those patients with glucose below 45mg/dL. That treatment will differentiate between those having stroke-like symptoms from hypoglycemia and those truly having a stroke.

However, it is less clear if dextrose should be routinely given to patients with mild coincidental hypoglycemia. A bolus administration of dextrose typically results in an acute, transient (less than one hour) elevation in serum glucose into a hyperglycemic range.^39^–^41^ The utility and safety of dextrose administration in patients with large focal neurological deficits but mild, possibly non-contributory hypoglycemia may need to be evaluated in the future. Hyperglycemia can also present as a stroke mimic, and elevated blood glucose on admission correlates with worse outcomes after stroke, specifically infarct expansion,^42^–^48^ and with intracranial hemorrhage after tPA.^49^,^50^

#### Current Prehospital Treatment Recommendation

##### Level A Recommendation

- Blood glucose should be checked in every patient with suspected stroke.
- Patients with hypoglycemia (glucose below 45mg/dL) should be treated with dextrose.

##### Level B Recommendation

- Stroke mimics are unlikely to be found in those hypoglycemic patients with a glucose of greater than 45mg/dL.

##### Level C Recommendation

- None given

### Supplemental Oxygen

#### Clinical Question

Does the prehospital administration of oxygen to patients with normal oxygen saturations improve outcomes in cases of suspected acute ischemic stroke?

#### Summary of Current Evidence

Every stroke patient should be assessed initially for airway compromise and treated accordingly. Airway compromise occurs more frequently in older patients, those with a severe stroke, or those with symptoms of dysphagia. Approximately 63% of patients with a hemiparetic stroke develop hypoxia.^51^

The evidence for oxygen use is less clear for normoxic patients. One randomized study compared the effect of 3L/min oxygen treatment for 24 hours versus no supplemental oxygen treatment on acute stroke patients and demonstrated no difference in survival and disability scores in those receiving oxygen.^52^ One a priori subgroup analysis of those with a more severe stroke demonstrated a statistically significant worsening of survival with supplemental oxygen. Several factors limited the conclusions of that study: a portion of the treated patients did not receive oxygen, patients had late time to therapy, and the study included hemorrhagic stroke patients. A more recent randomized trial with relatively few patients demonstrated short-term improvements but no long-term clinical differences between those given supplemental oxygen and those given no treatment.^53^ The research on this subject is limited.

Current practice for acute stroke patients includes the use of supplementary oxygen to maintain oxygen saturation above 94%.^32^,^54^ Beyond 94%, oxyhemoglobin is saturated and no further physiologic benefit is derived.

#### Current Prehospital Treatment Recommendation

##### Level A Recommendation

- None given

##### Level B Recommendation

- Oxygen should be delivered to a titrated dose of 94% oxygen saturation

##### Level C Recommendation

- None given

### Patient Positioning

#### Clinical Question

In what position should patients with possible strokes be transported?

#### Summary of Current Evidence

No clinical outcome studies exist to define the optimal position for transporting a patient with an acute stroke. A small number of studies evaluate blood flow and other secondary measures that might be useful in answering that question.

For patients with head injuries, setting the head of bed at 30 degrees alleviates elevated intracranial pressure.^55^,^56^ However, patients with strokes typically do not have elevated intracranial pressure. Cerebral blood flow and cerebral perfusion pressure both improved when the patient was put into the supine position.^57^,^58^ Mean flow velocity increased in patients with persistent occlusions when they were laid flat.^59^,^60^ The sitting position in patients who had suffered strokes caused reduced blood flow distal to the occlusion.^61^ When measured with tissue oxygenation index, cerebral oxygenation dropped in the upright patient and rose in the supine patient.^62^ Factors such as secretions, congestive heart failure, or respiratory distress frequently confound the acute stroke patient and preclude laying the patient flat because of effects on oxygen saturation and secretions. Oxygen saturation improved in stroke patients sitting upright, but that improvement was minimal.^63^,^64^ Positioning patients on their sides minimally affected oxygen saturation.^63^,^65^ Additionally, stroke patients frequently have sensory deficits in the laryngopharynx that can lead to aspiration.^66^ The evidence supports laying the head of the bed flat as tolerated in patients with suspected stroke.

#### Current Prehospital Treatment Recommendation

##### Level A Recommendation

- None given

##### Level B Recommendation

- None given

##### Level C Recommendation

- Patients should be laid flat as tolerated, unless precluded by clinical issues such as compromised respiratory status, secretions, or aspiration risk.

### 12-Lead ECG and Cardiac Monitoring

#### Clinical Question

Should a 12-lead ECG or cardiac monitoring routinely be performed in the prehospital setting for patients with suspected stroke?

#### Summary of Current Evidence

Cardiac monitoring detects significant cardiac pathology that can cause stroke or occur concurrent with stroke. Monitoring leads to earlier intervention. It is recommended in the prehospital setting and throughout the first 24 hours of care.^32^

Stroke patients frequently have cardiac arrhythmias or ECG abnormalities including ST segment depression, prolonged QTc interval, atrial fibrillation, T-wave inversion, conduction defects, premature ventricular beats, and left ventricular hypertrophy.^67^,^73^ One study showed ECG abnormalities in 60% of patients with cerebral infarction and 44% of patients with transient ischemic attack (TIA).^67^ In some of those events, such as atrial fibrillation, the cardiac event may have led to the stroke. In others, such as ST segment depressions, it is poorly understood why stroke patients develop ST segment depressions after their cerebral event. Atrial fibrillation, atrio-ventricular block, ST elevation, ST depression, and inverted T waves predicted mortality in patients with ischemic stroke.^67^,^72^ Care in units with cardiac monitoring led to improved outcomes at discharge, likely because of earlier intervention.^69^ The non-specific ECG changes do not change management in the prehospital setting, but significant arrhythmias may change management.

#### Current Prehospital Treatment Recommendation

##### Level A Recommendation

- None given

##### Level B Recommendation

- In patients with suspected stroke, a 12-lead ECG should be acquired and interpreted by prehospital or other emergency providers in a timely manner as long as it does not delay transport to a facility with tPA capabilities.

##### Level C Recommendation

- In a patient presenting with signs or symptoms of stroke and ST segment elevation myocardial infarction (STEMI), EMS should consider bypassing the nearest tPA capable facility for a facility with a catheterization lab.

### Fluid Assessment and Vascular Access

#### Clinical Question

Should normal saline be routinely given to patients with suspected stroke, and what type of vascular access should be attempted?

#### Summary of Current Evidence

No strong evidence supports or refutes routinely giving fluid boluses to stroke patients. Patients who have suffered a stroke are typically either euvolemic or hypovolemic.^32^ Hypotension occurs infrequently after stroke but leads to poor outcomes.^74^ A variety of hydration regimens on normotensive stroke patients resulted in no conclusive standard fluid regimen.^75^–^82^ A bolus of intravenous (IV) fluid acutely improved cerebral perfusion in focal ischemia from subarachnoid hemorrhage-induced vasospasm, a clinical scenario similar to ischemic stroke.^83^,^84^

It is useful to start a large bore IV access in any patient with a suspected stroke who may be receiving tPA and who could have subsequent hemorrhage. However, transport should not be delayed for this. Because of bleeding risk, multiple attempts at starting IV access should be limited. No studies negate or support the use of intraosseous access in stroke patients, but it is more invasive and carries theoretical greater risk of bleeding.

#### Current Prehospital Treatment Recommendation

##### Level A Recommendation

- None given

##### Level B Recommendation

- None given

##### Level C Recommendation

- Patients with low systolic blood pressure and no contraindications should be given a bolus of IV fluids.
- An IV should be placed as long as it does not delay transport and more than two attempts are not required.
- An IV should not be placed in the external jugular vein.

### Stroke Regionalization

#### Clinical Question

What parameters should be outlined in the stroke protocol to direct expeditious and appropriate transport? Should there be dispatch at high priority, documentation of time patient was “last seen normal,” limiting time on scene, hospital notification, transport to primary or comprehensive stroke center (CSC), and retriage from primary to CSCs?

#### Summary of Current Evidence

Early use of IV tPA is more effective at one hour than at three hours.^85^ It should not be used outside of the four and a half hour window. Recent AHA recommendations endorse the use of endovascular therapy after tPA for persistent LVOs.^86^ The efficacy of that therapy is also time sensitive. Thus, EMS protocols must guide timely evaluation and transport to appropriate facilities for those definitive interventions.

EMS should dispatch responders to suspected stroke patients with a high priority and attempt to shorten the time between the receipt of the call and the delivery of the patient to the emergency department. On initial history, responders must document “last seen normal time.”^32^ Use of specific language, rather than using the standard EMS run times, facilitates clear communication. Furthermore, paramedics can facilitate tPA delivery and definitive care by obtaining a medication list and pre-thrombolysis check list as well as the physician orders for life sustaining treatment (POLST). AHA recommends call to dispatch time of less than 90 seconds, EMS response time less than eight minutes, and an on-scene time less than 15 minutes.^32^ Higher priority of dispatch and hospital notification of a stroke both led to shorter times from ambulance call to arrival,^87^ assessment by a doctor,^4^ door to needle time,^57^,^87^–^91^ and door to imaging time.^22^,^88^,^92^–^95^ Patients received tPA more frequently at hospitals notified prior to patient arrival.^10^,^90^,^94^–^96^ Another study showed that one way to decrease on scene time was to explicitly direct an on-scene time of 15 minutes or less. That led to reductions in on-scene time over those with no instructions and those with general instructions to limit on scene time.^97^

A study published in 2010 concluded that 22% of people living in the continental U.S. have access to a primary stroke center (PSC) within 30 minutes, 43% have access within 45 minutes, and 55% have access within 60 minutes.^98^ Fewer patients have timely access to a CSC. Patients admitted to designated stroke centers versus community hospitals had increased tPA delivery rates.^99^ Additionally, admitting patients to designated stroke centers versus community hospitals was associated with increased tPA rates and decreased 30-day mortality.^100^ In a centralized model, where patients were transported to a stroke center preferentially over a community hospital, EMS transports occurred more frequently, they were given higher priority, more false positives were identified, more patients received tPA, and door-to-needle times were shorter.^101^,^102^

Throughout the U.S., more communities are shifting to a two-tiered system that includes PSCs and CSCs. Both assess for strokes and deliver tPA, but the CSCs also offer endovascular recanalization to patients with persistent LVOs. In light of the new AHA recommendations, that intervention is an evolving standard of care. The existence of both presents a transport dilemma to EMS. Should a patient with a suspected stroke be sent immediately to a CSC or initially to a PSC? If transport time to the CSC is longer, would it benefit the patient to go initially to the PSC to get tPA? As discussed above, some stroke scales can help to identify severity of stroke. In the future, these may direct transport decisions. The limited sensitivity and specificity of existing stroke scales may cause increased transport time in patients with a false positive on the stroke scale and delay in tPA administration for acute stroke patients with a false negative.

In California, hospitals sought PSC certification more frequently after counties developed protocols directing transport of patients with strokes to PSCs.^103^ EMS protocols indicating patients should go to PSCs may be beneficial for the patients and may also drive changes in hospital certification. A number of novel interventions such as Stroke Emergency Mobile Units or the incorporation of telemedicine may influence organization of stroke systems in the future. 104–128

#### Current Prehospital Treatment Recommendation

##### Level A Recommendation

- Time “last seen normal” should be documented.
- Suspected stroke patients should have a high-priority dispatch.
- Hospitals should be notified of a suspected stroke patient prior to arrival.

##### Level B Recommendation

- Scene times should be minimized and be 15 minutes or less if practical.
- Patients with a possible stroke should be transported to the nearest facility with tPA capabilities, preferably a PSC or CSC.

##### Level C Recommendation

- The integration of CSCs into EMS systems is rapidly evolving. Stroke systems should include formalized, rapid processes for higher level of care transports of patients with persistent LVOs to CSCs.

### Interfacility tPA

#### Clinical Question

Should tPA be delivered to patients by paramedics with confirmed strokes being transferred to CSCs for a higher level of care?

#### Summary of Current Evidence

The majority of acute stroke patients will be assessed and imaged at a PSC or community hospital. A subset of those patients will not respond to tPA and will require timely endovascular therapy at a CSC. The tPA infusion will need to be continued during transport. Recent studies indicate that this combination of tPA followed by endovascular intervention for persistent LVOs is rapidly becoming the standard of care.^86^

The use of prehospital tPA presents several logistical challenges. One study showed poor compliance with monitoring of blood pressure, delivery of antihypertensives and discontinuation of tPA with worsening neurological status. Despite these differences, there were similar neurological outcomes and intracranial hemorrhage rates between patients in whom guidelines were followed rigorously and those in whom they were not.^129^ Their mean transport time from PSC to CSC was 38 minutes +/−20 minutes.^129^ There are also logistical issues such as the implementation of infusion pumps in the field and the fact that tPA is not in the current scope of practice of paramedics in California.

Some areas are successfully sending nurses from the initial hospital with the patient and the tPA running in a hospital pump. That model avoids the complications of training paramedics in delivery of tPA and the use of new pumps. The practice is still evolving and requires further study.

#### Current Prehospital Treatment Recommendation

##### Level A Recommendation

- None given

##### Level B Recommendation

- None given

##### Level C Recommendation

- tPA should be initiated promptly on patients with confirmed strokes and no contraindications and, for persistent LVO, they should be transported as quickly as possible to a CSC for possible endovascular therapy.
- It is the responsibility of the sending physician to select appropriate means of transport and the appropriate level of the transporting staff.

## RESULTS

We reviewed protocols from all 33 LEMSAs within the state of California. Some LEMSAs had individualized stroke protocols, while others had stroke protocols embedded within those for altered mental status.

### Use of a Stroke Scale

Most (85%) LEMSAs directed the use of a stroke scale (See Table 1). The majority used CPSS. Of the 15% that did not specifically use a stroke scale, 9% recommended specific neurological exams that encompassed major key components of a stroke scale.

### Blood Glucose Evaluation

All LEMSAs recommended evaluation of blood glucose as part of their protocols for patients with suspected strokes (See Table 2). Seventy-three percent recommended a titrated dose of dextrose to correct low blood glucose. The titrated dose ranged from 60 to 80mg/dL.

### Supplemental Oxygen

Twenty-one percent of the LEMSAs advised routine use of oxygen regardless of oxygen saturation (See Table 3). Thirty-nine percent of LEMSAs advised a titrated dose of oxygen. The oxygenation goal of titration ranged from 94 to 100%.

### Patient Positioning

Three percent of LEMSAs recommend laying the head of bed flat as tolerated. Some (15%) recommend elevating the head of bed, and 21% recommend the lateral decubitus position (See Table 4).

### 12-lead ECG and Cardiac Monitoring

Fifty-eight percent of LEMSAs recommended cardiac monitoring, and 33% recommended a 12-lead ECG in patients with suspected strokes (See Table 5). Of those, some recommended both and some recommended one or the other.

### Normal Saline Administration or Fluid Assessment

Eighteen percent of LEMSAs recommended a normal saline bolus (See Table 6). Almost half (48%) recommended an IV line with minimal fluid. Twelve percent of LEMSAs gave direction about IV line location, gauge, or number of attempts.

### Stroke Regionalization

More than half (52%) of LEMSAs directed transport of patient to a stroke center. Eighty-eight percent recommended hospital notification from the field (See Table 7). Eighty-two percent of LEMSAs recommended documentation of duration of symptoms. Of those, most recommended documentation of “last seen normal.” Sixty-one percent of LEMSAs gave explicit directive to limit time on the scene, but only nine percent gave a specific time limit.

### Interfacility tPA

No LEMSAs commented on tPA during interfacility transport.

## CONCLUSION

Stroke is a complicated disease process. The science guiding optimal identification and treatment of stroke patients is evolving. Because of the difficulty in identifying stroke patients and the importance of their rapid transport to stroke centers, stroke presents a complex challenge for prehospital providers. The evidence-based recommendations presented in this paper will inform EMS medical directors and guide creation of protocols for identifying and treating stroke patients.

## Figures and Tables

**Table 1:** Use of a stroke scale.

LEMSA	Use of a stroke scale	Type of stroke scale	Emergent large vessel occlusion scale
Alameda County EMS agency	Yes	Cincinnati prehospital stroke scale	No
Central California EMS agency	No	N/A	No
City and County of San Francisco EMS agency	Yes	Cincinnati prehospital stroke scale	No
Coastal Valleys EMS agency	Yes	Cincinnati prehospital stroke scale	No
Contra Costa County	Yes	Cincinnati prehospital stroke scale	No
El Dorado County EMS agency	Yes	Cincinnati prehospital stroke scale	No
Imperial County EMS agency	Yes	LAPSS	
Inland EMS agency	Yes	Modified LAPSS	
Kern County EMS agency	Yes	Cincinnati prehospital stroke scale	No
Los Angeles County EMS agency	Yes	mLAPSS	No
Marin County EMS agency	Yes	Cincinnati prehospital stroke scale	No
Merced County EMS agency	No	N/A	No
Monterey County EMS agency	Yes	BEFAST	No
Mountain Valley EMS agency	Yes	Cincinnati prehospital stroke scale	No
Napa County EMS agency	Yes	Cincinnati prehospital stroke scale	No
Northern California EMS agency	Yes	Cincinnati prehospital stroke scale	No
North Coast EMS agency	No	Motor weakness, paralysis, speech distrubances, aphasia, headache, visual problems altered mental status	
Orange County EMS agency	No	No seizure prior to or during arrival, last seen normal within seven hours, GCS 10 or greater, and pronator drift or facial paresis	No
Riverside County EMS agency	Yes	Cincinnati prehospital stroke scale	No
Sacramento County EMS agency	Yes	Cincinnati prehospital stroke scale	No
San Benito County EMS agency	Yes	If <6 hours, Cincinnati prehospital stroke scale	No
San Diego County EMS agency	Yes	Cincinnati prehospital stroke scale	No
San Joaquin County EMS agency	Yes	Cincinnati prehospital stroke scale	No
San Luis Obispo County EMS agency	Yes	FAST	No
San Mateo County EMS agency	Yes	Cincinnati prehospital stroke scale	No
Santa Barabara County EMS agency	Yes	Cincinnati prehospital stroke scale	No
Santa Clara County EMS agency	Yes	Santa Clara County stroke scale - balance problems, diplopia, facial droop, arm drift, speech abnormalities, time last seen normal <6 hours	
Santa Cruz County EMS agency	Yes	Cincinnati prehospital stroke scale	No
Sierra-Sacramento EMS agency	Yes	Cincinnati prehospital stroke scale	No
Solano County EMS agency	Yes	Cincinnati prehospital stroke scale	No
Tuolumne County EMS agency	No	Weakness or paralysis on one side of the body/face, slurred speech, speech difficulty, difficulty with balance, inability to understand, difficulty in naming objects, confusion, difficulty swallowing, headache, visual disturbances (double vision, blindness, paralysis of extra-ocular muscles)	No
Ventura County EMS agency	Yes	Cincinnati prehospital stroke scale	No
Yolo County EMS agency	Yes	Cincinnati prehospital stroke scale	No
	85%		

*LEMSA,* local EMS agencies; EMS, emergency medical services; *LAPSS,* Los Angeles Prehospital Stroke Screen; *mLAPSS,* modified Los Angeles prehospital stroke screen; BEFAST, balance eyes face arm speech time; *GCS,* glasgow coma scale; FAST, face arm speech time

**Table 2:** Blood glucose in patients with suspected stroke. Glucagon dose in various prehospital protocols is listed in units milliliters and milligrams according to local protocol. We have copied them verbatim to demonstrate variation in presentation and practice.

LEMSA	Advise routine evaluation of BS	Advise titrated dose	Titration dose	Dextrose 10%	Notes
Alameda County EMS agency	Yes	No	N/A	Yes	
Central California EMS agency	Yes	Yes	80mg/dL with persistent AMS	Yes (25g IV)	
City and County of San Francisco EMS agency	Yes	Yes	60mg/dL	No	If BS<60 or known diabetic, dextrose 50%
Coastal Valleys EMS agency	Yes	Yes	60–80mg/dL	Yes (150mL IV of D10)	Glucagon 1mg IM if no IV access; recheck BS if symptoms not resolved; repeat additional dextrose 10% 100mL IV if glucose 60–80 or less; Dextrose 50% 25g IV if glucose 60–80 after 250mL Dextrose 10%. If Dextrose 50% unavailable, repeat Dextrose 10%.
Contra Costa County	Yes	Yes	60mg/dL	Yes	Check and treat if indicated
El Dorado County EMS agency	Yes	Yes	60mg/dL	No	25gm of 50% dextrose, if no IV then 1gm glucagon
Imperial County EMS agency	Yes	Yes	60mg/dL	No	Dextrose 50% 25gm IV or glucagon 1mg IM if no IV
Inland EMS agency	Yes	No	N/A	No	
Kern County EMS agency	Yes	Yes	60–80mg/dL	Yes	Use appropriate protocol to rule out narcosis/hypoglycemia then re-enter CVA protocol if indicated
Los Angeles County EMS agency	Yes	Yes	60mg/dL	No	Oral glucose if awake and alert, 50% 50mL, glucagon if no IV 1mg IM; if BS remains <60, repeat dextrose 50, repeat glucagon Q20min ×2
Marin County EMS agency	Yes	No	N/A	No	
Merced County EMS agency	Yes	Yes	75mg/dL	No	25gm IV if BS<75mg/dL, glucagon 1 U IM if no IV; repeat dextrose in 3–5 min if no response and continued hypoglycemia. Oral glucose if known diabetic and intact gag.
Monterey County EMS agency	Yes	Yes	70mg/dL	No	D50% 25gm IV if BS<70
Mountain Valley EMS agency	Yes	Yes	60mg/dL	No	25gms IV push; if BS<60mg/dL, repeat 1x; recheck BS in 5min after each dose; if no IV with BS<60, give glucagon 1U IM, may repeate 1x, recheck BG 5min after each dose
Napa County EMS agency	Yes	Yes	60mg/dL	Yes	Glucose paste 15gm PO if pt able to hold head upright, has gag reflex and can self-administer med; or D10% IV 25g 250mL or if no IV, D10% IO; if symptoms reverse and BS >60, slow D10% to remainder of dose; if no improvement after 5 minutes after D10% and BS still <60, give another D10% in 5g increments at 5–10min intervals reassessing BS levels and mental status every 5min
Northern California EMS agency	Yes	Yes	75mg/dL	No	Glucose paste po if suspected hypoglycemia, adequate gag reflex, hold head upright; check BS, then D50 up to 35 gm IV if BS<75; repeat 25 gm IV ×1 in 5min if BS still <75; if altered LOC and BS<75 and no IV, 1mg glucagon IM; no glucose if suspected CVA unless BS<75; if BS>250 treat with 500cc NS
North Coast EMS agency	Yes	No	N/A	No	
Orange County EMS agency	Yes	Yes	80mg/dL	No	Oral glucose if airway reflexes intact, 50% dextrose 50mL IV, may repeat ×1 if BS<80; glucagon 1mg IM if IV unable; IO ok for 50% dextrose if unable IV and no response to glucagon
Riverside County EMS agency	Yes	No	N/A	No	
Sacramento County EMS agency	Yes	No	N/A	No	
San Benito County EMS agency	Yes	Yes	70mg/dL	No	Treat as needed
San Diego County EMS agency	Yes	Yes	60mg/dL	No	If patient awake and gag, give 3 oral glucose tabs or paste (15g total); D50 25gm IV SO if BS<60; if pt remains symptomatic and BS remains <60 MR SO; if no IV, glucagon 1ml IM SO if BS<60
San Joaquin County EMS agency	Yes	Yes	60mg/dL	No	Paste if known diabetic, can hold head upright, can self-administer medication and has intact gag; If BS<60, then D50% 25gm or D10 50cc IV/IO bolus repeated every min until GCS 15; max dose D10 is 10cc/kg
San Luis Obispo County EMS agency	Yes	No	N/A	No	
San Mateo County EMS agency	Yes	Yes	80mg/dL	No	Avoid hyperglycemia
Santa Barabara County EMS agency	Yes	Yes	60mg/dL	Yes	If low BS suspected PO 15 g if BS<60, pt awake and able to swallow safely; if unable to swallow safely, glucagon IM 1 mg; if <60 and not able to swallow, D10W 25 mg IVP, glucagon if no IV; recheck BG 5 min after IV D bolus complete or 10 min after glucagon admin; if still <60, D10 IV 250 cc
Santa Clara County EMS agency	Yes	Yes	80mg/dL	No	If suspected hypoglycemia, 1 tube oral glucose paste, repeat in 5–15 min if no improvement; if BS<80, no oral/can’t oral D50 25 gm IVP; if no improvement, repeat dextrose or glucagon 1 mg IM; if no IV, and BS<80 and no improvement, glucagon 1mg IM
Santa Cruz County EMS agency	Yes	Yes	70mg/dL	No	Treat as needed
Sierra-Sacramento EMS agency	Yes	No	N/A	No	
Solano County EMS agency	Yes	Yes	60mg/dL	No	Treat hypoglycemia
Tuolumne County EMS agency	Yes	Yes	75mg/dL	No	25–50 gms IV push; 1 U IM glucagon if no IV access
Ventura County EMS agency	Yes	Yes	60mg/dL	Yes	If low BS suspected, PO 15 gm; If <60, D10W 10gm (preferred), D5W 10gm, D50W 12.5gm; Glucagon 1mg IM if no IV access Recheck BS 5 min after Dex or 10 min after glucagon; if still <60 D10W preferred or D5 or D50
Yolo County EMS agency	Yes	No	N/A	No	
	100%	73%		24%	

*LEMSA,* local EMS agencies; *BS,* blood sugar; *EMS,* emergency medical services; *IM,* intramuscular; *CVA,* cerbrovascular accident; *IV,* intravenous; *BG,* blood glucose; *PO,* per os (by mouth); *IO,* intraosseous infusion; *SO,* standing orders; *GCS,* glasgow coma scale

**Table 3:** Oxygen administration in patients with suspected stroke.

LEMSA	Advise routine use regardless of SpO2%	Advise titrated dose	Titration dose	Advise against with normal SpO2%	Notes
Alameda County EMS agency	No	Yes	94–99%	No	
Central California EMS agency	Yes	No	N/A	No	Low flow for suspected stroke (6L/min NC)
City and County of San Francisco EMS agency	No	No	N/A	No	Oxygen as indicated
Coastal Valleys EMS agency	No	Yes	94–98%	No	
Contra Costa County	No	Yes	94%	No	Low flow for BLS
El Dorado County EMS agency	No	No	N/A	No	Appropriate rate
Imperial County EMS agency	No	Yes	94%	Yes	
Inland EMS agency	No	No	N/A	No	
Kern County EMS agency	No	Yes	94%	No	Monitor/pulse oximetry
Los Angeles County EMS agency	No	No	N/A	No	As needed
Marin County EMS agency	No	No	N/A	No	
Merced County EMS agency	Yes	No	N/A	No	High flow, as tolerated
Monterey County EMS agency	No	No	No	No	Routine medical care
Mountain Valley EMS agency	No	No	N/A	No	As appropriate
Napa County EMS agency	No	Yes	94–97%	No	
Northern California EMS agency	Yes	No	N/A	No	
North Coast EMS agency	Yes	No	N/A	No	Oxygen therapy
Orange County EMS agency	No	Yes	95%	No	High flow mask if oxygen sat less than 95%
Riverside County EMS agency	No	No	N/A	No	
Sacramento County EMS agency	No	Yes	94%	No	Use lowest flow rate possible
San Benito County EMS agency	No	Yes	95%	No	Treat life threats
San Diego County EMS agency	No	Yes	94–98%	Yes	
San Joaquin County EMS agency	No	No	N/A	No	
San Luis Obispo County EMS agency	No	No	N/A	No	Evaluate for hypoxia
San Mateo County EMS agency	No	No	N/A	No	As indicated
Santa Barabara County EMS agency	Yes	No	N/A	No	High flow for spO2<95%, low flow for >95%
Santa Clara County EMS agency	No	No	N/A	No	
Santa Cruz County EMS agency	No	No	N/A	No	Treat life threats
Sierra-Sacramento EMS agency	Yes	Yes	94–100%	No	2L NC
Solano County EMS agency	Yes	No	N/A	No	High flow as tolerated
Tuolumne County EMS agency	No	No	N/A	No	As appropriate
Ventura County EMS agency	No	Yes	94%	No	
Yolo County EMS agency	No	Yes	94%	No	
	21%	39%			

*LEMSA,* local EMS agencies; *EMS,* emergency medical services; *NC,* nasal cannula; *BLS,* basic life support

**Table 4:** Patient positioning.

LEMSA	Recommend elevating head of bed	Lateral decubitus	Head of bed flat as tolerated	Notes
Alameda County EMS agency	No	No	Yes	Transport patient in supine position unless evidence of increasing ICP/intracranial hemorrhage, transport in semi fowlers with no more than 30 degrees head of bed elevation
Central California EMS agency	No	No	No	
City and County of San Francisco EMS agency	No	No	No	Position of comfort
Coastal Valleys EMS agency	No	No	No	
Contra Costa County	No	No	No	
El Dorado County EMS agency	No	No	No	
Imperial County EMS agency	Yes	Yes	No	
Inland EMS agency	No	No	No	
Kern County EMS agency	No	No	No	
Los Angeles County EMS agency	No	No	No	
Marin County EMS agency	No	No	No	
Merced County EMS agency	No	Yes	No	If not contraindicated by injuries, place patient in left lateral decubitus position
Monterey County EMS agency	No	No	No	
Mountain Valley EMS agency	No	No	No	
Napa County EMS agency	No	No	No	
Northern California EMS agency	Yes	No	No	30 degrees
North Coast EMS agency	Yes	Yes	No	Upright if gag reflex intact, left lateral with head elevated if gag reflex absent
Orange County EMS agency	No	No	No	
Riverside County EMS agency	No	No	No	Position patient as clinically indicated to meet physiologic requirements
Sacramento County EMS agency	No	No	No	
San Benito County EMS agency	No	Yes	No	Patients with depressed mentation or decreased gag reflex should be placed in a left lateral position
San Diego County EMS agency	No	Yes	No	If secretion problems place on affected side
San Joaquin County EMS agency	No	No	No	
San Luis Obispo County EMS agency	No	No	No	
San Mateo County EMS agency	Yes (unless spinal immobilization indicated)	No	No	
Santa Barabara County EMS agency	No	No	No	
Santa Clara County EMS agency	Yes	No	No	
Santa Cruz County EMS agency	No	Yes	No	If depressed mentation or decreased gag reflex
Sierra-Sacramento EMS agency	No	No	No	
Solano County EMS agency	No	Yes	No	Position of comfort, left lateral decubitus if vomiting
Tuolumne County EMS agency	No	No	No	
Ventura County EMS agency	No	No	No	
Yolo County EMS agency	No	No	No	
	15%	21%	3%	

*LEMSA,* local EMS agencies; *EMS,* emergency medical services; *ICP,* intracranial pressure

**Table 5:** 12 Lead ECG and cardiac monitoring in patients with suspected stoke.

LEMSA	Consider 12 Lead ECG	Advised cardiac monitoring	Notes
Alameda County EMS agency	Yes	No	Obtain 12-Lead ECG when a dysrhythmia or ACS symptoms are present (specifically watch for STEMI and/or A fib)
Central California EMS agency	No	Yes	Treat any arrhythmia
City and County of San Francisco EMS agency	No	No	
Coastal Valleys EMS agency	Yes (if possible)	No	
Contra Costa County	No	Yes	
El Dorado County EMS agency	No	No	
Imperial County EMS agency	Yes (consider)	Yes (consider)	
Inland EMS agency	Yes (consider)	No	
Kern County EMS agency	Yes	Yes	
Los Angeles County EMS agency	Yes (only if arrhythmia on monitor)	Yes	
Marin County EMS agency	No	No	
Merced County EMS agency	No	Yes	Treat rhythm as appropriate
Monterey County EMS agency	No	No	Routine medical care
Mountain Valley EMS agency	No	Yes	
Napa County EMS agency	Yes	No	Treat rhythm as appropriate
Northern California EMS agency	Yes (do not delay rapid transport)	Yes	
North Coast EMS agency	No	Yes	
Orange County EMS agency	No	Yes	
Riverside County EMS agency	No	No	
Sacramento County EMS agency	No	Yes	
San Benito County EMS agency	No	No	
San Diego County EMS agency	No	Yes	Monitor ECG
San Joaquin County EMS agency	No	Yes	ECG monitoring, Treat rhythm disturbances as appropriate
San Luis Obispo County EMS agency	Yes (consider)	No	
San Mateo County EMS agency	No	Yes	
Santa Barabara County EMS agency	No	Yes	
Santa Clara County EMS agency	No	No	Cardiac monitoring and ECG when medic suspects patient may have cardiac ischemia or any dysrhythmias
Santa Cruz County EMS agency	No	No	
Sierra-Sacramento EMS agency	Yes (if no delay in transport or patient care)	Yes	
Solano County EMS agency	No	Yes	
Tuolumne County EMS agency	No	Yes	ECG monitoring
Ventura County EMS agency	No	Yes	
Yolo County EMS agency	Yes	No	
	33%	58%	

*LEMSA,* local EMS agencies; *ECG,* echocardiogram; *EMS,* emergency medical services; *ACS,* acute coronary syndrome; *STEMI*, ST elevated myocardial infarction

**Table 6:** Normal saline administration.

LEMSA	Advise NS bolus	Advise defined bolus quantity	Advised TKO	Location	Notes
Alameda County EMS agency	No	No	Yes	Yes	No more than 1 AC attempt and no more than 2 IV attempts total, 18 GA, no smaller than 20 GA proximal to wrist, AC preferred
Central California EMS agency	No	No	Yes	No	
City and County of San Francisco EMS agency	No	No	Yes	No	NS TKO, If SBP<90 or poor perfusion, NS bolus
Coastal Valleys EMS agency	No	No	Yes	No	
Contra Costa County	Yes (If hypotensive or poorly perfused)	Yes (consider 250–500 cc if hypotensive)	Yes	No	
El Dorado County EMS agency	No	No	Yes	No	Twin cath or a second line is preferred for thrombolytic candidates. Limit IV attempts to two.
Imperial County EMS agency	No	No	No	No	IV prn
Inland EMS agency	No	No	No	No	Vascular Access
Kern County EMS agency	No	No	No	No	IV Line/Saline lock
Los Angeles County EMS agency	No	No	No	No	Venous access prn
Marin County EMS agency	No	No	No	No	
Merced County EMS agency	Yes	Yes (If SBP less than 90, then 500cc fluid boluses as indicated)	Yes	No	
Monterey County EMS agency	Yes (if appropriate)	No	No	No	
Mountain Valley EMS agency	No	No	Yes	No	
Napa County EMS agency	No	No	Yes	No	
Northern California EMS agency	Yes	No	Yes	No	Don’t delay rapid transport to establish IV, SBP at a minimum of 120mmHg, do not exceed 1.5L NS
North Coast EMS agency	No	No	Yes	No	
Orange County EMS agency	No	No	No	Avoid IO and EJ	
Riverside County EMS agency	No	No	No	No	
Sacramento County EMS agency	No	No	No	No	
San Benito County EMS agency	No	No	No	No	
San Diego County EMS agency	Yes	Yes	No	IV/IO adjust *prn*	250cc IV/IO with clear lungs to maintain BP≥120
San Joaquin County EMS agency	No	No	No	No	10cc/kg bolus if signs of shock present, max of 2L
San Luis Obispo County EMS agency	No	No	No	No	Establish vascular access
San Mateo County EMS agency	No	No	No	No	Consider IV/IO
Santa Barabara County EMS agency	Yes (500cc to keep SBP >100, Max 1L)	No	Yes	No	
Santa Clara County EMS agency	No	No	No		18G catheter minimum for CT scan, AC placement if possible. No more than 2 IV attempts
Santa Cruz County EMS agency	No	No	No	No	IVF if suspected shock
Sierra-Sacramento EMS agency	No (May bolus up to 1L)	No	Yes	No	
Solano County EMS agency	No	No	Yes	No	
Tuolumne County EMS agency	No	No	Yes	No	IV if HTN, in unstable IO OK if unable to gain IV access
Ventura County EMS agency	No	No	No	No	IV/IO access
Yolo County EMS agency	No	No	Yes	No	
	18%	9%	48%	12%	

*LEMSA,* local EMS agencies; *NS,* normal saline; *TKO,* to keep open; *EMS,* emergency medical services; *AC,* antecubital; *IV,* intravenous; *GA,* gauge; *SBP,* systolic blood pressure*; prn,* pro re nata (when necessary); *IO,* intraosseous infusion; *EJ,* external jugular; *BP,* blood pressure; *CT,* computed tomography; *IVF,* intravenous fluids; *HTN,* hypertension

**Table 7:** Stroke regionalization.

LEMSA	Advise documenting the duration of symptoms	Limit time on the scene	Transport to a stroke center	Hospital prenotification	Notes	Designated primary stroke centers	Comprehensive stroke centers	ReTriage from primary to comprehensive
Alameda County EMS agency	Yes (“Last seen normal”)	Yes	Yes	Yes		Yes	No	No
Central California EMS agency	Yes (“Last seen normal”)	Yes	No	Yes		No	No	No
City and County of San Francisco EMS agency	No	Yes	Yes	No	If potential stroke is suspected with symptoms for 4.5 hours or less, immediately transport patient to a designated Stroke receiving hospital	No	No	No
Coastal Valleys EMS agency	Yes (“Time of onset” or “last time patient known to be at baseline”)	Yes	No	Yes		No	No	No
Contra Costa County	Yes (“Last seen normal”)	Yes	Yes	Yes		Yes	No	No
El Dorado County EMS agency	Yes (“Time of onset”)	Yes (15 minutes)	Yes	Yes		No	No	No
Imperial County EMS agency	Yes (“Time of onset”)	Yes	No	Yes	Take patient to hospital with CT if suspected stroke and alert receiving hospital early.	No	No	No
Inland EMS agency	Yes (“Last seen normal”)	Yes	Yes (NSRC)	Yes	Transport Immediately	No	No	No
Kern County EMS agency	Yes (“Onset observed within 4 hours”)	No	Yes (appropriate facility)	Yes	Transport to appropriate facility in accordance with stroke policy	Yes	No	No
Los Angeles County EMS agency	Yes (“Time of sypmtom onset” and “last known well time”)	No	Yes	Yes		No	No	No
Marin County EMS agency	Yes (“Last known normal”	No	Yes	Yes	Call stroke if last seen normal <4 hours, rapid transport to patient’s preferred Primary Stroke Center, PSC as long as the estimated transport time is not more than 15 minutes longer than the nearest PSC; Preferred PSC: patient’s preference or PSC with patient’s medical records; No preferred PSC: transport to the closest PSC; Early Stroke Notification	No	No	No
Merced County EMS agency	No	Yes	No	No		No	No	No
Monterey County EMS agency	Yes (“Last known well”)	Yes (15 minutes)	Yes	Yes		No	No	No
Mountain Valley EMS agency	Yes (“Time of onset”)	No	No	No		No	No	No
Napa County EMS agency	Yes (“Time of onset”)	Yes	Yes (CVA receiving Center)	Yes		No	No	No
Northern California EMS agency	Yes (“Last seen normal”)	Yes	No	Yes		No	No	No
North Coast EMS agency	No	Yes	No	Yes	Transport code 3 if unconscious or conscious with progressive symptoms. Code 2, for others	No	No	No
Orange County EMS agency	Yes (“Time of onset”)	No	Yes	Yes		No	No	No
Riverside County EMS agency	Yes (“last known well”)	Yes (limit scene time to 10 minutes or less)	Yes	Yes		No	No	No
Sacramento County EMS agency	Yes (“Last observed to be normal”)	No	Yes (if CPSS>0 and time of onset 4 hours or less)	Yes		No	No	No
San Benito County EMS agency	Yes (“Time since symptoms onset/last time seen in premorbid state”)	No	No	Yes		No	No	No
San Diego County EMS agency	Yes (“Last time known normal”)	Yes	Yes	Yes	For suspected stroke with major deficit with onset of symptoms <4 hrs, expedite transport	Yes	No	No
San Joaquin County EMS agency	No	Yes	No	Yes		No	No	No
San Luis Obispo County EMS agency	Yes (“Last seen normal”)	No	No	Yes		No	No	No
San Mateo County EMS agency	Yes (“Time seen at Baseline”)	Yes (if symptoms present for <7 hrs)	No	Yes		No	No	No
Santa Barabara County EMS agency	Yes (“Last time seen normal”)	Yes	No	Yes	Consult with ED physician for further treatment measures	No	No	No
Santa Clara County EMS agency	Yes (“Last seen normal”)	No	Yes (if last seen normal <6 hrs)	Yes		No	No	No
Santa Cruz County EMS agency	Yes (“Time since symptoms onset/last time seen in premorbid state”)	No	No	Yes		No	No	No
Sierra-Sacramento EMS agency	Yes (“Time of onset of symptoms or when patient last seen normal”)	No	Yes (If symptoms <4 hours and within 30 min of stroke receiving center)	Yes		No	No	No
Solano County EMS agency	Yes (“Time of symptom onset”)	Yes	No	Yes		No	No	No
Tuolumne County EMS agency	No	No	No	No		No	No	No
Ventura County EMS agency	Yes (“Time last known well”)	Yes	Yes	Yes		No	No	No
Yolo County EMS agency	No	No	Yes (stroke symptoms less than or equal to 4.5 hours and within 45 min of stroke receiving center)	Yes		No	No	No
	82%	61%	52%	88%				

*LEMSA,* local EMS agencies; *EMS,* emergency medical services; *CT,* computed tomography; *NSRC,* neurovascular stroke receiving center; *CVA,* cerebrovascular accident; *PSC,* primary stroke center; *CPSS,* cincinnati prehospital stroke scale; *ED,* emergency department
